# Interictal Dysfunction of a Brainstem Descending Modulatory Center in Migraine Patients

**DOI:** 10.1371/journal.pone.0003799

**Published:** 2008-11-24

**Authors:** Eric A. Moulton, Rami Burstein, Shannon Tully, Richard Hargreaves, Lino Becerra, David Borsook

**Affiliations:** 1 P.A.I.N. Group, Brain Imaging Center, McLean Hospital, Harvard Medical School, Belmont, Massachusetts, United States of America; 2 Department of Psychiatry, Massachusetts General Hospital, Harvard Medical School, Boston, Massachusetts, United States of America; 3 Anaesthesia & Critical Care, Beth Israel Deaconess Medical Center, Harvard Medical School, Boston, Massachusetts, United States of America; 4 Imaging, Merck & Co. Inc., West Point, Pennsylvania, United States of America; 5 Department of Radiology, Athinoula Martinos Center for Bioengineering, Massachusetts General Hospital, Harvard Medical School, Charlestown, Massachusetts, United States of America; Duke Unviersity, United States of America

## Abstract

**Background:**

The brainstem contains descending circuitry that can modulate nociceptive processing (neural signals associated with pain) in the dorsal horn of the spinal cord and the medullary dorsal horn. In migraineurs, abnormal brainstem function during attacks suggest that dysfunction of descending modulation may facilitate migraine attacks, either by reducing descending inhibition or increasing facilitation. To determine whether a brainstem dysfunction could play a role in facilitating migraine attacks, we measured brainstem function in migraineurs when they were not having an attack (i.e. the interictal phase).

**Methods and Findings:**

Using fMRI (functional magnetic resonance imaging), we mapped brainstem activity to heat stimuli in 12 episodic migraine patients during the interictal phase. Separate scans were collected to measure responses to 41°C and noxious heat (pain threshold+1°C). Stimuli were either applied to the forehead on the affected side (as reported during an attack) or the dorsum of the hand. This was repeated in 12 age-gender-matched control subjects, and the side tested corresponded to that in the matched migraine patients. Nucleus cuneiformis (NCF), a component of brainstem pain modulatory circuits, appears to be hypofunctional in migraineurs. 3 out of the 4 thermal stimulus conditions showed significantly greater NCF activation in control subjects than the migraine patients.

**Conclusions:**

Altered descending modulation has been postulated to contribute to migraine, leading to loss of inhibition or enhanced facilitation resulting in hyperexcitability of trigeminovascular neurons. NCF function could potentially serve as a diagnostic measure in migraine patients, even when not experiencing an attack. This has important implications for the evaluation of therapies for migraine.

## Introduction

Defects in brainstem descending modulatory circuits may contribute to the onset of migraine, based on structural changes [1], [2] and functional abnormalities in brainstem areas during migraine attacks [3]–[5]. Enhanced responses in nociceptive spinal and trigeminal neurons could result from these abnormalities, which could reflect either unusually low levels of descending inhibition [6] or high levels of descending facilitation [7], [8]. In migraine, such hyperexcitability could lower the threshold of nociceptive neurons in response to meningeal inputs. If dysfunctional pain modulatory circuits exist in migraineurs, we hypothesized that functional changes should be evident in interictal (*i.e.* not experiencing an attack) migraine patients. Unlike previous studies of functional brainstem abnormalities in migraine, this study was able to directly compare migraine patients with healthy subjects due to the absence of ongoing migraine pain. Our results indicate that brainstem nucleus cuneiformis (NCF) is hypofunctional in migraine patients, possibly contributing to hyperexcitability of trigeminovascular neurons in migraineurs by either reduced descending inhibition or enhanced descending facilitation.

We compared brainstem responses to thermal stimuli in migraine patients when they were not having an attack and in healthy age-gender-matched control subjects. Until now, no study has provided direct evidence for specific functional changes that take place in the brainstem during the interictal migraine period. The major finding of this experiment was NCF hypofunction in interictal migraine patients relative to controls in response to perceptually similar thermal stimuli. NCF has previously been related to sensory modulation in animals [9]–[13] and humans [14]–[16]. Abnormal functioning during the interictal state provides further evidence that an altered endogenous system contributes to migraine pathophysiology.

## Methods

### Subjects

Migraine patients (9 females, 3 males; 42·2+/−11·7 years old) were free of neurological and other sensory dysfunctions, although two patients were taking antidepressants. The patients were selected by Dr. Burstein based on the criteria that they: (1) had acute intermittent migraine as defined by the International Headache Society (<14 attacks/month) and (2) had demonstrable allodynia during migraine attacks. For those patients taking daily medications (e.g., pre-emptive as opposed to medications to abort the attack), patients abstained from taking their migraine medications (Table S1) for one dosing interval prior to their scheduled scan session. Age- and gender-matched healthy subjects (9 females, 3 males; 42·3+/−11·9 years old) were also tested. This study was approved by the McLean Hospital Institutional Review Board, and met the scientific and ethical guidelines for human research of the Helsinki Accord (http://ohsr.od.nih.gov/guidelines/helsinki.html). All patients and subjects provided written informed consent to participate in this study.

Subjects were tested 7–10 days after their last attack, and were apparently not in the throes of a new migraine attack. Though patients were not surveyed days after their scan, the possibility that they could have an imminent impending attack seems unlikely for the following reasons: (1) no sensory differences were detected between the migraine and healthy subjects in this study; (2) the size of our interictal migraine group reduces the likelihood that the majority of these episodic migraine patients were about to have an attack, as attacks were relatively infrequent in this subject pool (<8 attacks/month); and (3) as part of another ongoing migraine imaging study, the patients were told to call us during their next migraine attack to schedule an impromptu imaging session, but did not call within the week following their scan. The ongoing migraine imaging study will compare central sensitization in the interictal vs. migraine attack state in a within-subject design.

### Stimuli

Temperatures were delivered using a 1·6×1·6 cm contact thermode (TSA-II, medoc Advanced Medical Systems, Ramat Yishai, Israel). Only the side of the face that was reported as sensitive during migraines by the patients was tested. The hand (dorsum) tested was on the same side as that for the face. The controls were matched to their corresponding migraine patient with regard to the side of the face tested.

Heat pain thresholds were determined using an ascending method of limits. Subjects were presented with a 32°C baseline temperature that increased 1°C/sec until they indicated their first detection of pain. Pain threshold was calculated as the average of three repetitions.

Functional scans began with 40 sec of the baseline temperature (32°C) followed by three 15 sec stimuli, each separated by 30 sec. The rate of temperature change was 4°C/sec.

### MRI scanning and image analysis

Imaging was conducted using a 3T Siemens Trio scanner with a quadrature head coil. Anatomical images were acquired with established imaging parameters [17]. For functional scans, a Gradient Echo (GE) echo planar imaging (EPI) sequence with TE/TR = 30/2500 was performed, with seventy-four volumes acquired. Each functional scan consisted of 33 slices oriented in an oblique plane to match the brainstem axis. This orientation of acquisition has proven useful for the functional imaging of brainstem structures [17]–[19]. Slices were 3·5 mm thick with in-plane resolution of 3·5 mm (64×64).

Functional imaging datasets were processed and analyzed using scripts within FSL 4·0 (FMRIB's Software Library, www.fmrib.ox.ac.uk/fsl) [20]. Image preprocessing was performed as previously described [17], with the exception that 5 mm FWHM spatial smoothing was used during preprocessing. First-level fMRI analysis of single subject data was performed using FMRI Expert Analysis Tool using FMRIB's Improved Linear Model (FEAT FILM) Version 5·4 with local autocorrelation correction [21]. Individual subjects were co-registered with respect to their brainstem for mixed-effect contrast group analysis of migraine vs. healthy subjects, and contrast maps were thresholded at z = 1·6 without correction for multiple comparisons. Single trial averages were calculated using in-house programs [22] in combination with functional time courses and an anatomically defined region of interest for NCF.

Other brainstem regions also involved in pain modulation (parabrachial nucleus and PAG) are located in close proximity to the NCF. However, we do not believe our activations involve the medial or lateral parabrachial nuclei for the following reasons: (1) our activation contrasts are in a similar location to those observed in other functional studies of healthy volunteers [14]–[16]; and (2) the parabrachial nuclei are located inferior to the NCF. We only observed dorsolateral PAG changes in one stimulus condition.

## Results

**Figure 1:**
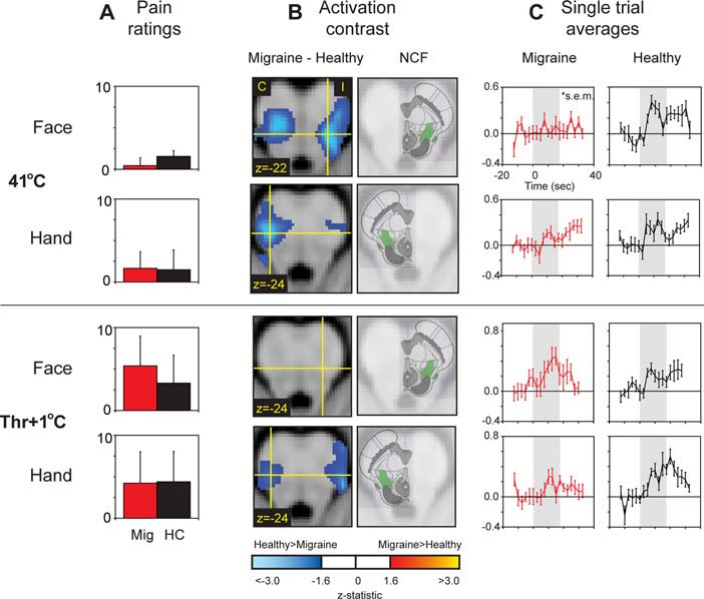
Functional differences in the brainstem and the brain of migraine vs. healthy control subjects. (A) Pain ratings: No significant effects of group or stimulation site for 41°C (2-Way repeated measures ANOVA [group, site], group F = 0·3646, p = 0·5521, df = 1, site F = 2·7355, p = 0·1123, df = 1) and Thr+1°C (2-Way repeated measures ANOVA [group, site], group F = 0·2918, p = 0·5950, df = 1, site F = 0·4060, p = 0·5312, df = 1). Mig = migraine subject; HC = healthy control. (B) Activation contrast: Interictal migraine subjects show decreased nucleus cuneiformis (NCF) responses to stimuli relative to controls. The exception is Thr+1°C on the face. The green area in the reference images highlight NCF (adapted from Duvernoy[46]). C = contralateral to stimulus site; I = ipsilateral. p<0·05 (uncorrected). (C) Single trial averages: Responses recorded from anatomically defined NCF region of interest. Y-axis = normalized % signal change. Gray shade represents stimulus application. N = 12 in each condition, except for Thr+1°C for Face (N = 11). One patient was excluded because of stimulus-correlated motion; the corresponding control was also excluded.

For the 41°C stimulus, the area of the dorsolateral pons centered on the NCF showed significantly greater activation in control subjects than the migraine patients (Fig. 1). This area of the brainstem, albeit with less spatial resolution, has previously been found active during migraine attacks in positron emission tomography studies [3], [5]. Activation in NCF was observed bilaterally for the face stimulation site, and was predominantly contralateral for the hand (Table 1). In both cases, inspection of the group activation map for healthy subjects revealed significant activation in this area, whereas migraine patients did not show significant activation. In addition, trial averages that relate to the timing of the stimuli show distinguishable temporal responses in the healthy controls (Fig. 1). Pain intensity ratings (0 = no pain; 10 = max pain) to the 41°C stimulus were not significantly different between the migraine and control groups (Fig. 1).

**Table 1:** Summary of group activation contrasts of interictal migraine vs. control within the NCF.

Stimulus	Site	Side	Peak z-statistic	Peak z-stat MNI152 x,y,z	Volume (mm^3^)
41°C	Face	C	−2.9	10,−28,−22	160
		I	−2.9	−8,−32,−22	272
	Hand	C	−2.7	10,−28,−22	104
		I	−1.8	−10,−26,−22	32
Thr+1°C	Face	C	-	-	-
		I	−1.6	−6,−28,−16	8
	Hand	C	−2.2	14,−26,−14	192
		I	−1.7	−8,−28,−11	48

C = Contralateral to affected side.

I = Ipsilateral to affected side.

NCF ROI volume = 648 mm^3^.

The noxious heat stimulus (Thr+1°C) applied to the face and hand resulted in significant activation of this same NCF region in both migraine and control subjects. At this higher intensity level, the NCF activation in the control subjects was significantly greater than in migraine subjects for stimuli applied to the hand, though not the face (Fig. 1; Table 1). The average temperature applied was 47±3°C, and was not significantly different for the migraine and control groups, and neither for the face and hand (2-Way repeated measures ANOVA [group, site], group F = 0·7814, p = 0·3872, df = 1, site F = 0·1677, p = 0·6865, df = 1). Pain intensity ratings were not significantly different between the groups or stimulation sites (Fig. 1).

## Discussion

The decreased relative activation in NCF of the migraine group suggests a dysfunction in this brainstem descending pain modulatory system. NCF sends dense neural projections to the rostroventral medulla [23], which directly modulates dorsal horn nociceptive transmission neurons in the spinal cord and in the analogous medullary dorsal horn [6], [24]. NCF receives reciprocal input from lamina I dorsal horn neurons [25]–[27], which may drive its activation and thus complete a possible negative-feedback loop [15]. Inputs to the NCF from higher brain structures related to modulatory processing may also contribute to NCF output [6], [15], [28]. In the healthy group, the NCF activation we observed with both 41°C and noxious heat could be conceivably driven by thermal and nociceptive inputs from lamina I in the trigeminal nucleus (face)/dorsal horn (hand) [26], as well as wide dynamic range neurons in deeper lamina such as IV and V [29].

In addition to the aforementioned structural connectivity data, functional investigations of NCF are suggestive of a role in descending pain modulation. Animal and human studies have suggested that the NCF can inhibit and facilitate nociception through cholinergic and glutamatergic mechanisms, which may be triggered by noxious stimulation, central sensitization, as well as the expectation of pain. In animal studies, electrical stimulation of NCF produces opioid-mediated analgesia through excitatory cholinergic projections to the nucleus raphe magnus (NRM) [9], [11]. Additionally, electrical stimulation or microinjection of morphine into NCF in the rat can also activate glutamatergic projections to the NRM, leading to modulation of pain-related behaviors [10], [13]. In addition to the NCF-NRM pathway, recent evidence suggests that morphine microinjection into NCF may activate a compensatory descending modulatory pathway when the NRM is lesioned [12]. Human imaging studies have reported that activations in NCF and the rostroventral medulla are correlated during repeated noxious stimulation [14], and also that NCF may activate during punctate mechanical hyperalgesia, suggesting that it is involved in a alterations in descending pain modulation [16]. NCF is also activated during the expectation of pain, indicating that awareness of impending pain can trigger a preparatory modulatory process in NCF [15].

We interpret our results as follows: NCF hypofunction is a characteristic of migraine sufferers that is detectable during their interictal phase, and reflects a dysfunction of descending modulation. The dysfunction we observed may be a result of damage secondary to a history of repeated migraine attacks [30]–[32]. However, the mechanism of such damage is not known although a neurovascular etiology has been suggested. Though the hypofunction we observed intuitively suggests a decrease in inhibition, the NCF is an integrative structure that contains both ‘off’ and ‘on’ cells [33], and a decrease in the overall level of activation would not rule out the presence of enhanced facilitation. The hyperexcitability of nociceptive circuitry downstream of the NCF may contribute to central sensitization at the onset of migraine. NCF dysfunction could thus lead to progressive changes in the spinal trigeminal nucleus (localized allodynia), and/or the thalamus and spinal cord (generalized allodynia). However, note that while NCF hypofunction was found in migraineurs during their interictal phase, the NCF dysfunction may change or even be supplanted by abnormalities in other brain regions during the different phases of an actual migraine attack.

The absence of a difference between migraine and control subject NCF activation for noxious stimuli to the face in migraine patients (Fig. 1) is unexpected, in light of the impaired descending modulation model. We speculate that the NCF dysfunction manifests only when insufficient afferent drive is present to fully trigger the descending modulatory system. In other words, increased afferent drive may be required to overcome the NCF dysfunction to activate descending inhibition. In migraine patients, noxious heat applied to the face may more effectively activate descending modulatory circuitry relative to the control group, whereas non-noxious heat did not seem to activate descending inhibition to the same extent. That this speculative intensity-dependent relationship appears to be specific to the face could be attributed to the involvement of potentially sensitized trigeminal afferents in migraine patients. At higher intensity levels than used in this study, increased afferent drive may also recruit activation of other modulatory centers (e.g., PAG). The existence of such an intensity-dependent activation of the descending modulatory system is mere conjecture at this point.

Why would a non-noxious stimulus (41°C) activate a brainstem modulatory region like the NCF? Several possible explanations may account for this: (1) 41°C is known to activate nociceptors, and what we observe may reflect an enhancement of their sensitivity; (2) since NCF sends descending projections to second-order wide dynamic range neurons [29], which encode both non-noxious and noxious stimulus intensities, perhaps NCF also affects innocuous stimulus processing at sub-perceptual levels in the migraine interictal phase; (3) low-threshold C-fiber afferents may convey other information that differentially activate or involve descending modulatory systems [34], including the NCF; (4) corticobulbar input in migraine patients may differentially modulate NCF activity [35] regardless of stimulus intensity via a sensory-cognitive mechanism; (5) the interictal migraine brain may be hyper-excitable in regards to general sensory processing [36], which may or may not include the involvement of NCF. These explanations expand the possible role of descending modulatory processing in migraine.

How is it possible to see differences in modulatory circuitry without seeing a difference in pain sensitivity between migraine and healthy subjects using comparable stimuli? While we do not know the specific answer to this question, there are a number of possible explanations. NCF can be triggered during the anticipation of pain, before a stimulus is even applied [15], [37]: this suggests that the perception of physical stimuli is not necessary for NCF activation, and that cognitive processes may influence NCF activation in healthy subjects perhaps more effectively than in migraine patients. In addition, structural changes detected in this region of the brainstem [1], [2], [38], which includes white matter changes, may alter the coupling of the blood-oxygen-level- dependent response and neural activity [39]. The latter interpretation leaves open the possibility that NCF in interictal migraine patients may function perfectly well, but that the fMRI signal itself is dissociated with neural activity in this area. Finally, patient medications may have dampened the fMRI signal, given that healthy controls were free of medications. However, this possibility is perhaps less likely given that: (1) eight out of twelve patients were not taking pre-emptive medications for their migraine, (2) patients discontinued their medications for one dosage cycle prior to imaging, (3) the significant changes observed were specifically localized to NCF, and were not global as might be expected for a drug, and (4) the heterogeneity of the medications taken by the patients reduces the likelihood of a mass action of any one pharmacological mechanism influencing the fMRI signal. An important caveat is that intermittent use of abortive migraine medications may have unknown long term effects on nociceptive processing.

We expected to observe changes in the PAG given its involvement in descending modulation during migraine attacks [3]. The relationship between the NCF and PAG in descending modulation is incompletely understood, but they share cytoarchitectural homology [6] and are extensively connected [35], [40]. However, only in one condition (Hand Thr+1°C) did we observe hypofunction in the dorsolateral PAG (data not shown). We interpret this negative result as an indication that PAG may only be recruited in descending modulation with higher levels of pain, such as that experienced during migraine.

We propose that NCF hypofunction in migraine patients contributes to central sensitization during attacks through partial loss of inhibition and/or enhanced facilitation of ascending nociceptive pathways. A prevailing theory to explain the occurrence of migraine attacks is that hyperexcitability develops along the trigeminovascular pathway [41]. Disruption of a descending modulatory system in migraine patients could cause such hyperexcitability, and has been previously hypothesized to be an underlying cause for migraine pathology [5], [41]–[43]. While this state of putative disinhibition/facilitation did not appear to impact the perception of thermal stimuli during the interictal phase, we hypothesize that it may facilitate central sensitization at the onset of migraine. In our migraine patients, we found that NCF activation was disrupted not only for stimuli applied to the face, but also the hand. This relationship suggests that for migraineurs with extended allodynia, the disinhibition/facilitation of the encoding of noxious heat that accompanies NCF dysfunction is not specific to the head, but may be generalized to other parts of the body.

Currently, drugs that are developed to treat migraine have focused on alleviating symptoms during a migraine attack. However, migraine patients have shown abnormalities in cortical sensory processing even between migraine attacks. Using innocuous and noxious thermal stimuli as test stimuli, we have identified specific brain structures that are hypofunctional in migraine patients when they are not having an attack. This model may be a useful surrogate in evaluating pre-emptive or disease modifying therapies for migraine patients. Considering that recurrent episodic migraines appear to transform into severe daily headache [44], this brainstem dysfunction could be used to evaluate treatments to prevent this transformation [45]. Future studies should evaluate the potential of current and future migraine preventative agents to correct this dysfunction, thereby improving descending inhibition and reducing headache frequency.

## Supporting Information

Table S1 Patient medications
(0.06 MB DOC)
