# Supplementary material for: Interictal Dysfunction of a Brainstem Descending Modulatory Center in Migraine Patients
Source: PLoS One. 2008 Nov 24;3(11):e3799. doi: 10.1371/journal.pone.0003799 (PMC2582961; doi:10.1371/journal.pone.0003799)
Supplement: Table S1 — Patient medications (0.06 MB DOC) [file pone.0003799.s001.doc]

**Table S1. Patient medications.**

| Patient # | Abortive Rx | Pre-emptive Rx | Analgesic | Other |
| --- | --- | --- | --- | --- |
| 1 | Naratriptan (Amerge) | - | Indomethicin (Indocin) | - |
|  |  |  | Acetaminophen/Aspirin/Caffeine |  |
|  |  |  | (Excedrin Migraine) |  |
| 2 | Zolmitriptan (Zomig) | Amitriptyline | Excedrin Migraine | - |
| 3 | Rizatriptan(Maxalt) | - | Acetaminophen (Advil) | - |
| 4 | Sumatriptan (Imitrex) | - | Hydrocone/Ibuprofen (Vicoprofen) | Tizanidine (Zanaflex) |
| 5 | Zolmitriptan (Zomig) | Propranolol | - | - |
| 6 | Sumatriptan (Imitrex) | Prozac | - | - |
| 7 | Rizatriptan (Maxalt) | - | - | Levothyroxine sodium (Levoxyl) |
| 8 | Sumatriptan (Imitrex) | - | - | Oral contraception |
| 9 | Sumatriptan (Imitrex) | - | Excedrin Migraine | Levothyroxine (Synthroid) |
|  |  |  |  | Venlafaxine (Effexor) |
| 10 | - | - | Ibuprofen (Advil) | Ranitidine (Zantac) |
|  |  |  |  | Nifedipine (Adalat) |
|  |  |  |  | Valsartan (Diovan) |
| 11 | Rizatriptan (Maxalt) | - | Excedrin Migraine | Albuterol (Proventil) |
|  |  |  |  | Fluticasone/Salmetrol (Advair) |
|  |  |  |  | Venlafaxine (Effexor) |
|  |  |  |  | Methylphenidate (Ritalin) |
| 12 | Sumatriptan (Imitrex) | Verapamil (Calan) | - | - |

*Patients abstained from pre-emptive medications for one dosage cycle prior to the scan session.
